# Neural Network Prediction of Keratoconus in AIPL1-Linked Leber Congenital Amaurosis: A Proof-of-Concept Pilot Study

**DOI:** 10.3390/jcm14186499

**Published:** 2025-09-15

**Authors:** Daniel R. Chow, Raheem Remtulla, Glenda Vargas, Goreth Leite, Robert K. Koenekoop

**Affiliations:** 1 Faculty of Medicine and Health Sciences, McGill University, Montreal, QC H3G 2M1, Canada; daniel.chow@mail.mcgill.ca; 2 Department of Ophthalmology & Visual Sciences, Faculty of Medicine and Health Sciences, McGill University, Montreal, QC H3G 2M1, Canada; raheem.remtulla@mail.mcgill.ca; 3 McGill University Health Center (MUHC) Research Institute, Montreal, QC H3G 2M1, Canada; 4 Departments of Pediatric Surgery, Human Genetics, and Adult Ophthalmology, McGill University Health Center, Montreal, QC H3G 2M1, Canada

**Keywords:** Leber congenital amaurosis, retinal degeneration, gene therapy, inherited retinal disease

## Abstract

**Background/Objectives:** Keratoconus (KC) can rapidly erode vision in children with Leber congenital amaurosis (LCA), yet screening usually depends on costly corneal imaging that is often unavailable. We evaluated whether a lightweight, image-free neural network fed only routine clinical and genetic variables can detect KC in patients with AIPL1-related LCA. **Methods:** This retrospective, proof-of-concept pilot study analyzed chart data for 19 children with biallelic AIPL1 mutations (6 with KC) seen at five tertiary eye centers between January and December 2004. Ten baseline predictors were entered into a feed-forward neural network. Records were randomly split 60/20/20 into training, validation and test sets; 20 replicate networks were trained. The mean test accuracy, sensitivity and specificity across runs were the primary outcomes. **Results:** The ensemble achieved a mean test accuracy of 91.6% (SD 12.8%), sensitivity of 87.5% (SD 13.1%) and specificity of 93.5% (SD 17.0%). A total of 6 of the 20 runs made no test-set errors, and 16 achieved 100% specificity. The median training time per network was less than 1 s on a laptop CPU. **Conclusions:** This exploratory pilot shows that a point-of-care, image-free neural network using readily available clinical and genetic data accurately identified KC in AIPL1-LCA. External validation in larger, contemporary cohorts is warranted, but the approach could help triage scarce imaging resources and enable timely corneal–collagen cross-linking in settings where tomography is inaccessible.

## 1. Introduction

Keratoconus (KC) is a progressive corneal ectasia that can reduce best-corrected visual acuity to counting-fingers levels by the third decade if undetected [1]. Advanced KC and other corneal opacities account for 3–5% of global blindness [2]. Children with Leber congenital amaurosis (LCA) carry a disproportionate risk: a masked school survey found KC in 29% of LCA pupils [3], and 25% of those with biallelic AIPL1 variants develop KC during childhood, likely amplified by the oculo-digital eye-rubbing phenomenon [4]. Progression is swift, with 80% of untreated pediatric eyes worsening within three years [5], and 10–15% eventually need keratoplasty. Fortunately, early corneal–collagen cross-linking (CXL) halts ectasia in upwards of 80% of young corneas [6].

KC screening depends on Scheimpflug or OCT tomography, but a single device costs tens of thousands of dollars and often yields unusable scans in LCA because of nystagmus and photophobia [7]. Although the FDA-cleared IDx-DR system shows that AI can deliver autonomous eye-care triage [8], a 2023 Cochrane review confirmed no non-imaging risk calculators for KC [9]. We therefore evaluated whether ten routine demographic, clinical and genetic variables, including AIPL1 status, could power a lightweight neural network to flag KC at a child’s first visit, directing scarce imaging and cross-linking to those who need them most. Recent pediatric studies report high diagnostic accuracy for AI-assisted retinopathy-of-prematurity screening [10], smartphone-image triage of common disorders [11], and deep-learning strabismus classification [12], illustrating how lightweight models are reshaping children’s eye care. We selected a shallow feed-forward neural network because preliminary five-fold cross-validation indicated that it captured clinically plausible non-linear relations among genotype and ocular variables and produced a modest improvement in discrimination over penalized logistic regression, while L2 regularization curbed over-fitting.

## 2. Materials and Methods

### 2.1. Design and Ethics

We conducted a retrospective, proof-of-concept diagnostic accuracy test using the de-identified cohort described by Dharmaraj et al. [4]. The McGill University Health Centre REB waived consent. The procedures followed the Declaration of Helsinki.

### 2.2. Participants

Charts from 26 children with molecularly confirmed AIPL1-LCA were reviewed: 7 lacked KC status, leaving 19 patients. Previous corneal surgery or any missing predictor value also triggered exclusion (Table 1).

### 2.3. Reference Standard

KC was classified as present or absent by a fellowship-trained cornea specialist using Scheimpflug tomography or slit-lamp criteria in the source study. These determinations were accepted unchanged.

### 2.4. Predictors

Ten variables available at the first specialist visit were evaluated: age, region of origin, visual acuity category, photophobia, nyctalopia, oculo-digital phenomenon, maculopathy, optic nerve pallor, pigmentary retinopathy, and biallelic AIPL1 mutation status (AIPL1 mutation status was coded as present/absent for biallelic variants, with W278Stop as the most common mutation in this cohort). Table 1 summarizes the baseline demographic and ocular characteristics.

### 2.5. Data Preprocessing and Partitioning

Categorical predictors were one-hot encoded, and continuous variables were z-score normalised. Records with any missing field were list-wise deleted. The data was stratified 60%/20%/20% into training, validation and test sets while preserving KC prevalence.

### 2.6. Model Development

**Figure 1:**
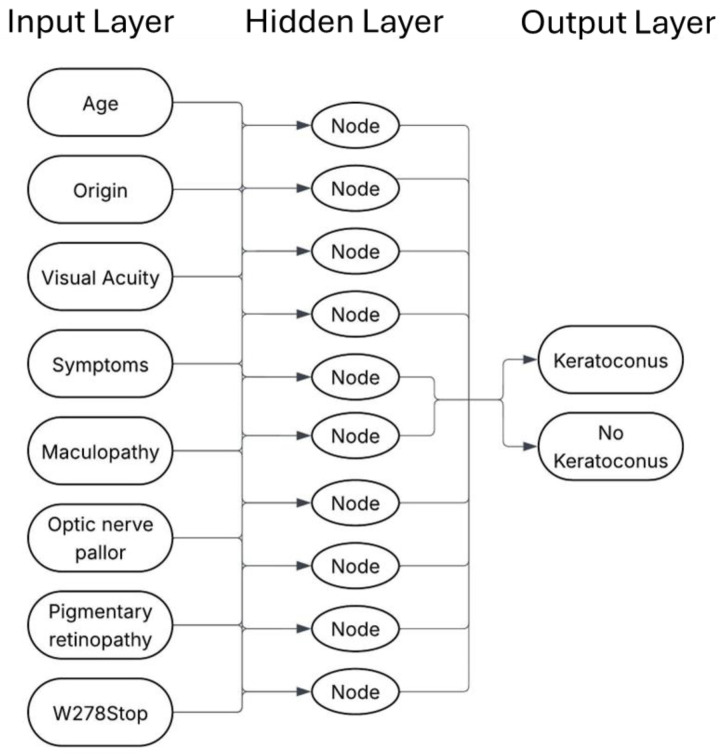
Network architecture.

The final model comprised a single hidden layer with 10 neurons (10-4-1 topology) using sigmoid activations and Xavier initialization (see Figure 1). The training minimized the binary cross-entropy, a method well-suited to imbalanced, binary-outcome data, via the scaled conjugate gradient optimizer (learning-rate, 0.01; momentum, 0.9) with an L2 regularization coefficient of 0.01. The training stopped when the validation loss failed to improve or after 1000 epochs, whichever occurred first. Full-batch updates (all 19 records) were used. Performance estimates were obtained with five-fold stratified cross-validation repeated 20 times. Twenty replicate networks were trained, each initialized with a unique random seed under the same scaled conjugate gradient procedure in MATLAB R2024a (MathWorks, Natick, MA, USA). Categorical AIPL1 genotypes were one-hot encoded, and continuous variables were z-standardized. A full MATLAB script and JSON file of the trained weights are available from the corresponding author on reasonable request.

### 2.7. Evaluation

The accuracy, sensitivity, specificity and F1-score were averaged across the 20 runs. The entire pipeline was repeated after omitting the AIPL1 variable (“drop-column” analysis) to gauge its incremental value, and paired two-tailed t tests were performed to compare the overall accuracies with α = 0.05. Performance metrics (accuracy, sensitivity, specificity and F1-score) were averaged over 20 independent stratified five-fold cross-validation repeats to minimize the stochastic variation intrinsic to small samples. Given the pilot sample size, formal feature-attribution analyses (e.g., SHAP or permutation importance) were deferred to avoid over-interpreting noisy estimates; these will be undertaken once a larger, multi-gene cohort is assembled.

### 2.8. Reporting

Every effort was made to align with the TRIPOD-AI guidelines. The completed TRIPOD-AI checklist is available in the Supplementary Materials [13].

## 3. Results

Nineteen children with AIPL1-LCA (mean age, 16 ± 13.7 y; 63% <18 y) were analyzed; six (31.6%) already had KC and were older than their KC-negative peers (25.7 ± 16.9 vs. 11.1 ± 9.1 y). Maculopathy and pigmentary retinopathy were more frequent in KC eyes (Table 1).

**Figure 2:**
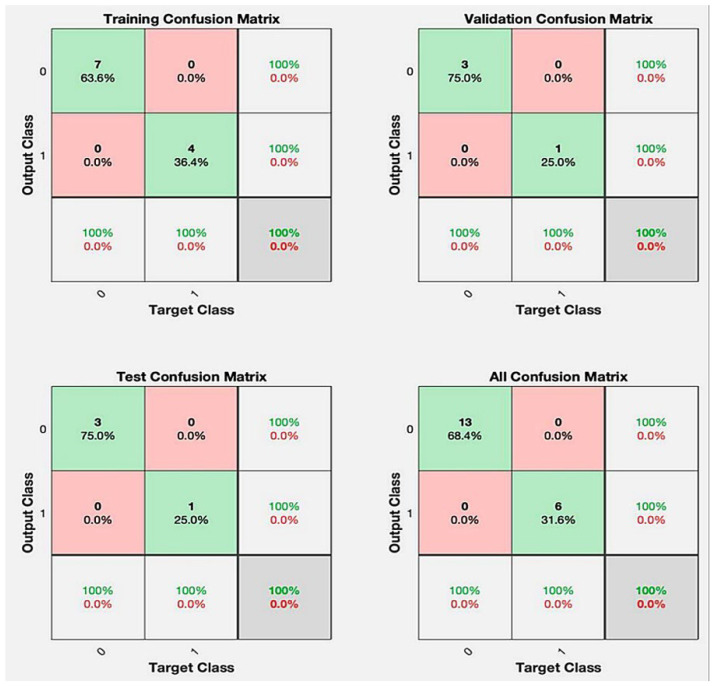
Confusion matrices of a representative best run. Four 2 × 2 matrices summarize the predictions for the training, validation, test and overall datasets (true positives/negatives in green; false positives/negatives in pink). Across 20 random initializations, 6 of 20 runs had no test-set errors and 16 of 20 achieved 100% specificity, demonstrating the robustness of the neural network.

With all ten baseline predictors, including genotype, the 20-run neural-network ensemble achieved a mean accuracy of 91.6 ± 12.8%, sensitivity of 87.5 ± 13.1%, specificity of 93.5 ± 17.0% (standard deviations from 20 repeat five-fold cross-validation runs in this pilot cohort) and F1-score of 0.901 (Table 2). Six runs made no test-set errors. A confusion matrix is provided in Figure 2.

Removing the AIPL1 variable reduced the performance to an accuracy of 80.8 ± 17.8%, sensitivity of 75.0 ± 33.6%, specificity of 83.5 ± 19.0% and F1-score of 0.781 (Table 2). The validation–loss curves also diverged earlier, suggesting incipient over-fitting when genotype was absent.

**Figure 3:**
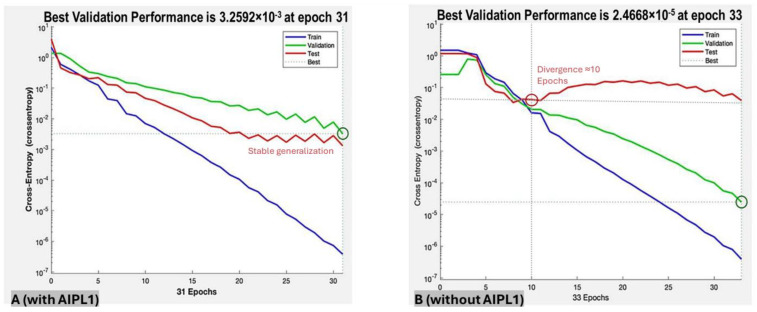
Training and validation loss curves for the neural network, showing stable generalization with AIPL1 genotype (**A**) and over-fitting without it (**B**). Cross-entropy loss (log_10_ scale) across 31 epochs for the training set (blue), the validation set (green) and the test set (red); the dashed green trace marks the best validation performance, with the circled point indicating the epoch selected for testing. (**A**) Model with AIPL1-genotype input: red and green curves converge and plateau together, showing stable generalization. (**B**) Model without genotype input: validation (green) and test (red) losses plateau together at ≈1 × 10^−1^ cross-entropy after ≈10 epochs, signaling early over-fitting.

Overall, supplying genotype information improved all four performance indices, chiefly by lowering false-negative errors, and maintained parallel training–validation losses to the end of training (see Figure 3).

## 4. Discussion

This lightweight, image-free neural network combines ten routine variables with a binary AIPL1 genotype flag and classifies KC in AIPL1-LCA with 92% accuracy, 88% sensitivity and 94% specificity (the 95% CIs are wide given N = 19; see Table 2’s foot-note). Because inference runs offline in milliseconds, risk can be conveyed during the first genetics visit, when Scheimpflug or anterior-segment OCT is often impracticable. Our proof-of-concept network complements recently reported pediatric AI tools for retinopathy-of-prematurity screening, smartphone-based ocular triage and automated strabismus classification [10,11,12] because it runs entirely on routinely charted variables; such lightweight models can triage children in low-resource clinics and channel scarce imaging or cross-linking to those at greatest risk.

Most published KC AI models are tomography-centric: convolutional networks that examine Placido or Scheimpflug maps reach 88–97% accuracy for established KC and 76–92% for subclinical disease [14]. Purely clinical calculators are almost absent; the only prior model, trained in adults, achieved an AUROC of 0.81 [15]. Our tool therefore fills a recognized gap and, like the FDA-cleared autonomous diabetic retinopathy detector [16], shows that data-light approaches can still deliver high diagnostic performance.

Roughly one-quarter of children with biallelic AIPL1 variants develop rapidly progressive KC [3,5]. In many low-resource settings, tomography is unavailable or yields unusable scans [9]. A bedside score that identifies high-risk eyes could prompt timely CXL, optimize scarce imaging and improve cost effectiveness. First-line pediatric CXL is estimated to cost 3174 GBP per quality-adjusted life year [17]. Prioritizing those most likely to progress should enhance that ratio. The model runs on any laptop or within an electronic record, supporting use in both tertiary and outreach clinics alike.

Drop-column analysis confirmed the contribution of genetics. Indeed, adding AIPL1 status improved the specificity by 10% and overall accuracy by 10.8%. Truncating AIPL1 variants have long been linked to anterior ectasia and keratoglobus [4], plausibly via stromal collagen fragility or vigorous oculo-digital rubbing [18]. Photophobia was the most influential non-genetic predictor, echoing mechanobiological hypotheses that sustained squinting increases tangential corneal stress [9]. The physiological plausibility of these weights argues against over-fitting. Because optic nerve pallor appeared in close to 79% of our cohort, the same architecture could, with larger datasets, be retrained to grade pallor or predict other LCA-related morbidities such as early cataract or pigmentary retinopathy. Although the present pilot centers on AIPL1, an identical network can be re-trained for other LCA genotypes, such as CEP290, GUCY2D, RPE65 or CRX, simply by adding the corresponding binary genotype flags and fine-tuning on larger, multicenter datasets, thereby extending its utility to broader pediatric populations.

### Limitations

Several methodological constraints qualify these findings. These performance standard deviations are consistent with the pilot cohort size and will shrink proportionally as larger, multicenter datasets are incorporated. Averaging metrics across 20 repeat stratified five-fold cross-validation runs already dampens the run-to-run volatility, yet confirmation in larger multicenter cohorts will be required to cement the robustness. Likewise, feature importance plots were not generated for this 19-patient pilot; larger external datasets will be required before such interpretability metrics can be reported with confidence. Because AIPL1-LCA is uncommon (5–7% of all LCA) [4], the nineteen-patient sample confers wide confidence intervals and limits the assessment of more complex model architectures. Synthetic minority over-sampling and other data augmentation techniques will be evaluated once a larger, multicenter dataset, including raw imaging, is available. Applying them to the present 19-patient tabular cohort risks information leakage and the inflation of performance estimates. The retrospective design yielded uneven documentation; behavioral cofactors such as eye rubbing, atopy and sleep quality were often absent, reducing the explanatory power. The KC classification relied on expert slit-lamp or Scheimpflug impressions, and quantitative indices such as K_max_ or ABCD staging were unavailable for direct benchmarking. The cohort drew cases from five centers across four continents, which enhances the external relevance but may conceal local imaging and referral biases. Prospective, harmonized multicenter validation remains necessary before clinical adoption. We are now assembling a prospective, harmonized multicenter cohort to externally validate, recalibrate and extend the model. The expanded cohort will also permit statistically sound feature importance analysis (e.g., SHAP) and the safe application of data augmentation methods to boost model generalizability.

Despite these shortcomings, the project provides preliminary evidence that a low-footprint, non-imaging neural network can help triage LCA children at highest risk for KC.

## 5. Conclusions

A lightweight, image-free neural network can flag LCA children carrying pathogenic AIPL1 variants who are at high risk of KC with up to 92% accuracy. Because it runs offline in milliseconds, the tool could guide timely referral for CXL even where tomography is unavailable, potentially preserving vision and improving cost-effectiveness. Validation in larger, prospective cohorts is the next step before clinical adoption.

## Figures and Tables

**Table 1:** Baseline demographic and ocular characteristics of 19 LCA patients, stratified by KC status.

Predictor (Model Input)	All Patients (N = 19)	KC-Converters (N = 6)	Non-Converters (N = 13)	Absolute Difference (KC-Non)
Age, years	16.0 ± 13.7	25.7 ± 16.9	11.1 ± 9.1	+14.6 yr
South Asian origin	5 (26%)	4 (67%)	1 (8%)	+59 pp
Severe Visual Impairment (all patients) *	19 (100%)	6 (100%)	13 (100%)	0 pp
Photophobia (yes)	5 (26%)	2 (33%)	3 (23%)	+10 pp
Nyctalopia (yes)	16 (84%)	5 (83%)	11 (85%)	−1 pp
Oculo-digital phenomenon (yes)	2 (11%)	1 (17%)	1 (8%)	+9 pp
Maculopathy (yes)	19 (100%)	6 (100%)	13 (100%)	0 pp
Optic nerve pallor (yes)	15 (79%)	6 (100%)	9 (69%)	+31 pp
Pigmentary retinopathy (yes)	17 (89%)	6 (100%)	11 (85%)	+15 pp
W278Stop mutation present	11 (58%)	3 (50%)	8 (62%)	−12 pp

* BCVA was collapsed into a three-level ordinal scale (0 ≥ 20/200; 1 = 20/200–20/400; 2 = Counting Fingers/Hand Motion/Light Perception/No Light Perception).

**Table 2:** Performance metrics with vs. without AIPL1 genetic information.

	Dataset with AIPL1				Dataset Without AIPL1			
	Accuracy	Specificity	Sensitivity	F1-Score	Accuracy	Specificity	Sensitivity	F1-Score
Training	95.5 (14.3)	95.5 (16.9)	96.3 (9.3)		84.5 (21.5)	85.0 (20.0)	84.0 (22.0)	
Validation	85.0 (17.0)	87.1 (26.6)	66.7 (45.4)		82.5 (18.3)	83.0 (19.0)	82.0 (20.0)	
Test	87.5 (22.2)	92.1 (20.5)	86.9 (29.4)		68.8 (24.2)	70.8 (28.6)	62.2 (48.6)	
Overall	91.6 (12.8)	93.5 (17.0)	87.5 (13.1)	0.901	80.8 (17.8)	83.5 (19.0)	75.0 (33.6)	0.781

Note. Exact 95% confidence intervals are not displayed because the pilot sample (19 children; 6 KC-positive) yields very wide bounds; values should be interpreted accordingly.

## Data Availability

De-identified individual participant data that underlie the results reported in this article (predictor variables, outcome label), the MATLAB training script, and the final network weights will be made available to qualified researchers on reasonable request from the corresponding author (robert.koenekoop@mcgill.ca) beginning at the time of publication. Requestors must supply a brief study protocol and sign a data use agreement stating that the data will be used only for non-commercial research and that no attempt will be made to re-identify participants. No additional supporting documents are available.

## Supplementary Materials

The following supporting information can be downloaded at: https://www.mdpi.com/article/10.3390/jcm14186499/s1, File S1: TRIPOD-AI Checklist: completed reporting checklist [19,20].

## Abbreviations

The following abbreviations are used in this manuscript:

AIPL1	aryl hydrocarbon receptor-interacting protein-like 1 gene
CXL	corneal–collagen cross-linking
KC	keratoconus
LCA	Leber congenital amaurosis
OCT	optical coherence tomography
REB	Research Ethics Board
